# Comparative analysis of differences for pathological upgrade and incomplete resection in endoscopic snare papillectomy for ampullary adenomas: A single-institution retrospective study

**DOI:** 10.1371/journal.pone.0330220

**Published:** 2025-08-12

**Authors:** Xin Chen, Jie Huang, Xiaoli Chen, Qingqing Luo, Min Gao, Jiaguo Wu, Xingkang He

**Affiliations:** 1 Department of Gastroenterology, Sir Run Run Shaw Hospital, Zhejiang University School of Medicine, Hangzhou, China; 2 Department of Endoscopy Center, Sir Run Run Shaw Hospital, Zhejiang University School of Medicine, Hangzhou, China; Hokkaido University: Hokkaido Daigaku, JAPAN

## Abstract

**Background:**

Inconsistent pathological diagnoses between pre- and post-endoscopic snare papillectomy (ESP) biopsies were frequently observed. We aimed to compare the differences in pathological upgrade and incomplete resection between endoscopic snare papillectomy for ampullary adenomas.

**Methods:**

The included patients were those referred to Sir Run Run Shaw Hospital and underwent ESP for an ampullary adenoma between 2012 and 2022. The endoscopic and clinicopathological features of ampullary adenomas were obtained using white light endoscopy, narrow-band imaging endoscopy, and endoscopic ultrasound (EUS). Adverse events, histological diagnosis, and follow-up data were also collected.

**Results:**

Overall, 40 patients underwent ESP of ampullary adenomas and were included in the study. Seventeen patients had inconsistent pre- and post-ESP pathological diagnoses, as they were upgraded from either low-grade dysplasia (LGD) to high-grade dysplasia (HGD) or from HGD to adenocarcinoma. Various characteristics varied between the pathological upgrade and non-upgrade groups, such as alanine transaminase (ALT), alkaline phosphatase levels (ALP), erosion and redness of papilla, a hybrid histological type, procedure time and extended lower bile duct width identified through EUS. Differences were observed between the complete and incomplete resection groups in terms of ALT, Gamma-glutamyl transferase (GGT) levels, tumor extension into the bile duct, and width of lower bile duct extension as determined by EUS.

**Conclusions:**

Pathological upgrading were relatively common after ESP for ampullary adenomas. Preoperative identification of specific clinical and endoscopic features can enhance diagnostic accuracy and inform treatment strategies.

## Introduction

Benign ampullary lesions are considered relatively rare and often occur sporadically without specific symptoms [1]. In contrast, ampullary adenomas are a common type of benign but pre-cancerous lesion arising from the duodenal papilla [2,3]. According to autopsy studies, the prevalence of ampullary adenomas has been estimated to be approximately 0.04% to 0.12% [4,5]. Due to the increasing number of endoscopies being performed, the detection rate of ampullary adenomas has also increased over the past several years [6]. According to existing data, patients with familial adenomatous polyposis almost always present with duodenal adenomas and have a high risk for ampullary carcinoma [7–9]. Previously, radical surgery methods have been the standard approach for the treatment of ampullary adenomas, including transduodenal surgical ampullectomy, pancreas-preserving duodenectomy, and pancreaticoduodenectomy [10]. Considering the high postoperative morbidity and mortality rates of these procedures, endoscopic advances have shifted the paradigm of treatment towards endoscopic papillectomy, which is less invasive and associated with lower morbidity [11].

Several studies have demonstrated the high feasibility and safety of endoscopic papillectomy for ampullary adenomas and early-stage carcinomas, with success rates ranging from 46 to 92% [12–17]. Long-term follow-up data have also confirmed the safety and efficacy of endoscopic papillectomy for ampullary adenomas with acceptable morbidity [18,19]. In a recent retrospective study, endoscopic papillectomy demonstrated safety and lower R0 rates compared to transduodenal surgical ampullectomy. Endoscopic papillectomy, despite exhibiting elevated rates of recurrences and retreatments, demonstrated non-inferiority to the surgical therapy in relation to overall survival [20]. The choice between endoscopic and surgical approaches poses uncertainty and complexity, necessitating a thorough and precise assessment of ampullary lesions prior to clinical decision-making. The final diagnosis of a benign or malignant lesion is based on its histology, which is imperative for determining the best therapeutic approach. Performing a preoperative biopsy of the suspected lesion is crucial for confirming the presence of an adenoma. However, the forceps biopsy technique may not take a representative sample of the lesion and may instead miss foci of adenocarcinoma within adenomatous tissue [21]. Therefore, the value of pre-procedural biopsies is a matter for discussion in clinical practice. Bellizzi et al. reported poor diagnostic agreement (64%) between biopsies and resected specimens [22]. It is quite often that the pathological underestimation of ampullary tumors has occurred during preoperative biopsies, and further surgical treatment has sometimes been required.

This study aimed to investigate the differences in clinical, biochemical, and endoscopic features between pathological upgrading and incomplete resection in patients who underwent endoscopic snare papillectomy (ESP) for ampullary adenomas. We hoped that our study could provide some information on the underestimation of the severity of ampullary adenomas by biopsy and help optimize patient selection for ESP.

## Methods

### Study design and patients

A retrospective study was conducted on patients undergoing endoscopic snare papillectomy (ESP) for ampullary lesions at Sir Run Run Shaw Hospital from 2012 to 2022. All data were fully anonymized and accessed on July 10, 2023. The inclusion criteria were as follows: (1) lesions at the papillary duodenum were confirmed by gastroduodenoscopy and endoscopic ultrasonography and were recorded as having a diameter of less than 4 cm [23]. (2) The T1 stage was confirmed by endoscopic ultrasound (EUS), with less than 2 cm of protrusion into the bile duct or pancreatic duct. Lymph node metastasis was ruled out by contrast-enhanced computed tomography (CT) and/or magnetic resonance imaging (MRI). (3) The pathological diagnosis of an adenoma was made and excluded the presence of malignancy. The exclusion criteria were as follows: (1) T2 stage (invasion of the duodenal muscularis propria) or above, (2) suspected lymph node metastasis or distant metastasis, (3) preoperative, pathological confirmation of malignancy, (4) presence of familial adenomatous polyposis (FAP) or adenoma with periampullary laterally spreading tumor (LST), (5) ampullary adenomas that had laterally spread on the duodenal wall, (6) there was poor cardiopulmonary function that would not be able to tolerate the endoscopy procedure, (7) patient refusal of endoscopic therapy or participation in follow-up. All inclusion and exclusion criteria were applied to the pre-EP status of the patients. Written informed consent was obtained from all patients before the procedure. This study was approved by the local institutional review board. The information that was collected included patient demographics, the levels of alanine transaminase (ALT), aspartate transaminase (AST), alkaline phosphatase (ALP), gamma-glutamyl transferase (GGT), carbohydrate antigen 19-9 (CA199), the appearance of ampullary lesions under white light endoscopy, and the results of narrow-band imaging and endoscopic ultrasound. ALT and AST elevation were specified as being ≥2 times the upper limit of normal (ULN), whereas ALP and GGT elevation were indicated as ≥1.5 times the ULN. The upper limit of normal values utilized in our laboratory were as follows: ALT: 50 U/L, AST: 40 U/L, ALP: 125 U/L, GGT: 60 U/L. In narrow-band imaging (NBI) of the gastrointestinal tract, a demarcation line (DL) distinguishes between cancerous and non-cancerous tissue by highlighting enhanced mucosal and vascular patterns. The presence of the White Opaque Substance (WOS), a white substance that is opaque to endoscopic light, is occasionally observed within the epithelium during NBI with magnifying endoscopy. The tumor size was assessed using EUS by measuring its longest axis. Tumor extension into the bile duct was detected during EUS as a hypoechoic or moderate echoic lesion that extended past the papillary orifice. The width of the lower bile duct was measured at the distal common bile duct during EUS. The follow-up period was 6–60 months. Pathological upgrading was defined as an upgrade from the biopsy pathological diagnosis to the final histopathological diagnosis of the excised tumor. Pathological diagnosis was made by same group of experienced pathologists with no notable alterations in diagnostic thresholds or classification systems during the study period. This study received written ethics approval from Ethics Committee of Zhejiang University School of Medicine Sir Run Run Shaw Hospital (Approval NO. 20230367). The informed consent could be exempted according to the ethics committee approval letter.

### The endoscopic papillectomy procedure

ESP was performed by a single experienced endoscopist (JiaGuo Wu) on each patient. EUS was routinely performed prior to ESP. After a full evaluation, we used a duodenoscope to carry out ESP using an appropriately sized polypectomy snare (chosen from three different diameters of 13 mm, 27 mm (Boston Scientific, New York, USA), and 30 mm (JiuHong Medical Instrument Co. Ltd., Changzhou, China)) with the aim of performing resection *en-bloc*. Submucosal injection was performed when the lesion edge could not be clearly exposed. Piecemeal resection was preferred in cases of large ampullary adenomas that required more than one resection. The blended endocut mode (ERBE, Tübingen, Germany) was always used for ESP. Carbon dioxide insufflation was routinely used during ESP. An indomethacin suppository was used routinely before the procedure. Prophylactic antibiotics were administered. Drainage of the pancreatic duct was attempted by inserting a 5 Fr pancreatic stent (5 cm long) only if the pancreatic duct intubation was successful. Bile duct drainage with a 7Fr stent (5 or 7 cm long) with or without biliary sphincterotomy was also attempted. After complete resection of the lesion, we used clips to fully close the wound and prevent bleeding in patients who had successfully placed pancreatic duct and biliary duct stents. Residual or recurrent adenomatous tissue was treated either with argon plasma coagulation (APC) (ERBE, Tübingen, Germany), another type of snare resection, or radiofrequency ablation. Complete endoscopic resection was defined as the complete excision of the lesion with negative horizontal margins (HM) and vertical margins (VM), regardless of the number of sessions required. Incomplete endoscopic resection was defined as the inability to completely remove the lesion, either with positive HM or positive VM, regardless of the number of sessions required.

### Adverse events and surveillance

The adverse events of ESP included in this study were pancreatitis, perforation, and bleeding. Surveillance after endoscopic papillectomy consisted of endoscopic review after 1, 3, 6, and 12 months during the first year and every 12 months thereafter. Post-endoscopic acute pancreatitis was defined according to the revised Atlanta classification, requiring at least two of the following: (1) new or worsened abdominal pain consistent with pancreatitis; (2) serum amylase elevation ≥3 times the upper limit of normal within 24 hours after ESP; (3) imaging findings (e.g., pancreatic edema or inflammation on CT) when available [24]. As per the updated Atlanta classification, mild acute pancreatitis is defined by the lack of organ failure and the absence of local or systemic complications [24]. In our cohort, serum amylase levels were routinely measured 24 hours post-procedure in all patients. Diagnoses were based on both clinical and biochemical criteria, with imaging confirmation in selected cases.

### Statistical analysis

The data were expressed as the means and standard deviations. Chi-square analysis or Fisher’s exact test was used to compare proportions. The Student’s *t*-test or nonparametric Wilcoxon rank-sum test was used to compare differences among continuous variables. Although we acknowledged the significance of multiple testing correction, this study was structured as an exploratory analysis. To address the trade-off between type I and type II errors, we contemplated utilizing the Benjamini-Hochberg procedure to manage the issue within the framework of limited sample sizes and numerous comparisons. A *P*-value <0.05 was considered statistically significant. All analyses were performed using R language software (version 4.1) (R Foundation for Statistical Computing, Vienna, Austria).

## Results

### Characteristics of the included patients and ampullary adenomas

**Fig 1:**
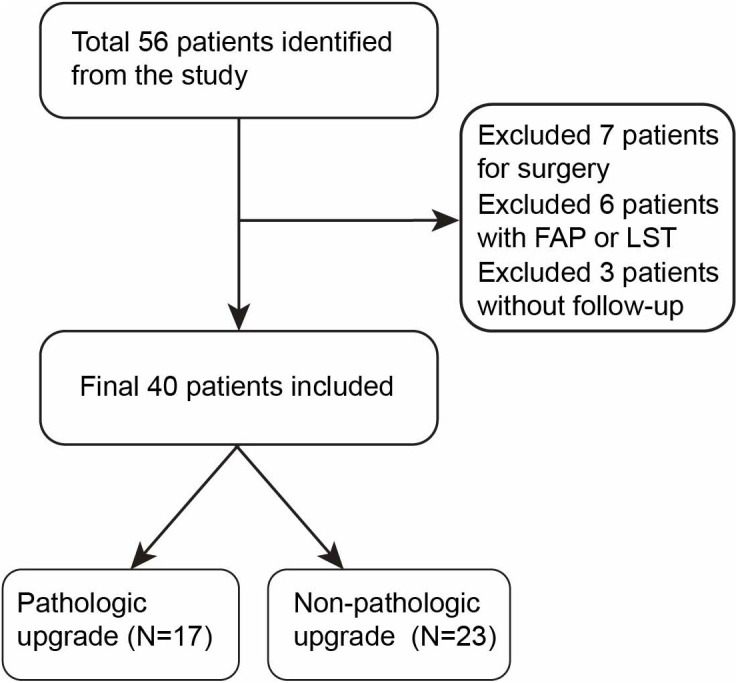
Study population. FAP, familial adenomatous polyposis; LST, laterally spreading tumor.

From 2012 to 2022, a total of 56 patients with ampullary lesions were referred to our hospital for endoscopic papillectomy. Sixteen patients were excluded from the study according to our inclusion and exclusion criteria (surgery, N = 7; non-availability of follow-up, N = 3; FAP or adenoma with periampullary LST, N = 6). A detailed flowchart is shown in Fig 1. The mean age of the included patients was 59 years. There were 23 males (57.5%) and 17 females (42.5%). More than half of the patients were found to be asymptomatic at presentation (26/40). The most common presenting complaint in symptomatic patients was abdominal pain (11/14, 78.6%). The mean size of the ampullary lesion was 19 mm (ranging from 6 to 35 mm), based on a pathological specimen measurement after the endoscopic papillectomy. Overall, complete endoscopic papillectomy of ampullary lesions was achieved in 29 of the 40 patients (72.5%) during the initial attempt. Three patients were referred for surgical intervention due to incomplete resection.

### Pathological characteristics obtained by the preoperative and postoperative examination of lesions

The distribution of pathological diagnoses among preoperative biopsies and endoscopic resection samples is summarized in Table 1. Of the pathological diagnoses of preoperative biopsies, 35 patients had ampullary adenomas with low-grade dysplasia (LGD), 4 patients had ampullary adenoma with high-grade dysplasia (HGD), and 1 patient had a neuroendocrine tumor. According to the pathological diagnosis of samples after ESP, 22 patients had adenomas with LGD, 10 patients had adenomas with HGD, 7 patients had adenocarcinoma, and 1 patient had a neuroendocrine tumor. Nine patients were upgraded from LGD to HGD, 4 patients were upgraded from LGD to adenocarcinoma, 3 patients with HGD were upgraded to adenocarcinoma, and 1 patient with a T1 stage neuroendocrine tumor was upgraded to the T2 stage. In total, 17 patients underwent pathological upgrading.

**Table 1:** Distribution of pathological diagnosis between pre-endoscopic snare papillectomy (ESP) and post-ESP.

Pathological diagnosis	Pre-ESP	Post-ESP
Low-grade dysplasia	35	22
High-grade dysplasia	4	10
Adenocarcinoma	0	7
Neuroendocrine tumor	1	1

ESP: endoscopic snare papillectomy.

### Characteristic differences in upgraded pathological examinations

Compared to the non-pathological upgrade group, the levels of ALT and ALP were greater in the group with pathological upgrading. There were no differences in age or gender ratio between the two groups. Under white light microscopy, erosion and redness of the papilla, as well as the width of the lower bile duct detected by EUS, were associated with pathological upgrading. In terms of morphological classification, the hybrid histological type was associated with pathological upgrade, while the periampullary histological type was associated with non-upgrade. In NBI, the presence of a white opaque substance covering the lesion and a demarcation line of the lesion were not significantly associated with pathological upgrading. Echo features of EUS and the extension of the tumor into the bile duct under EUS were also not significantly associated with pathological upgrading. The procedure time was significantly associated with pathological upgrading, while number of preoperative biopsies was not related with pathological upgrading. Some significant findings retained their significance following the application of the Benjamini-Hochberg method for multiple comparisons (Table 2).

**Table 2:** Comparative differences between upgrade and non-upgrade pathological diagnosis of ampullary adenomas.

Characteristics	Non-upgrade (N = 23)	Upgrade (N = 17)	P value	Corrected p-values
Age (years)	56.9	61.8	0.25	/
Gender				
Male	12(52.2%)	11(64.7%)	0.64	/
Female	11 (47.8%)	6 (35.3%)		
ALT				
Normal	22(95.7%)	10 (58.8%)	0.01	**0.02**
Elevate	1 (4.3%)	7 (41.2%)		
AST				
Normal	23(100.0%)	13(76.5%)	0.06	/
Elevate	0 (0.0%)	4(23.5%)		
ALP				
Normal	23(100.0%)	11(64.7%)	<0.01	**0.01**
Elevate	0 (0.0%)	6(35.3%)		
GCT				
Normal	19(82.6%)	9(52.9%)	0.09	/
Elevate	4(17.4%)	8(47.1%)		
Total bilirubin			0.06	/
Normal	20(87.0%)	13(76.5%)		
Elevate	3(13.0%)	4(23.5%)		
CA199			0.06	/
Normal	23(100.0%)	13(76.5%)		
Elevate	0(0.0%)	4(23.5%)		
Erosion and redness of papilla			<0.01	**0.01**
Yes	2(8.7%)	9(52.9%)		
No	21(91.3%)	8(47.1%)		
Morphological classification			<0.01	**0.01**
Intra-ampullary	2(8.7%)	3(17.6%)		
Periampullary	17(73.9%)	1(5.9%)		
Hybrid	4 (17.4%)	13 (76.5%)		
WOS (NBI)			0.8	/
Yes	14 (60.9%)	12(70.6%)		
No	6(26.1%)	3 (17.6%)		
NA	3(13.0%)	2(11.8%)		
DL (NBI)			0.6	/
Yes	16(69.6%)	9(52.9%)		
No	2 (8.7%)	2(11.8%)		
NA	5(21.7%)	6 (35.3%)		
EUS picture			0.1	/
Hypoechoic lesion	12(52.2%)	14(82.4%)		
Moderate echoic lesion	11(47.8%)	3 (17.6%)		
Tumor extends into the bile duct under EUS			0.2	/
Yes	1(4%)	4 (23.5%)		
No	22 (96.0%)	13 (76.5%)		
Tumor size under EUS (cm)	1.64	1.67	0.9	/
Width of lower bile duct under EUS(cm)	0.61	0.93	0.02	**0.02**
Number of preoperative biopsies	4.3	3.5	0.06	**/**
Procedure time (min)	36.8	52.3	<0.01	**0.01**
Complication			0.99	/
Yes	6(26.1%)	5(29.4%)		
No	17(73.9%)	12(70.6%)		

ALT, Alanine transaminase; AST, Aspartate transaminase; ALP, Alkaline phosphatase; GGT, Gamma-glutamyl transferase; CA199, Carbohydrate antigen 19−9; NBI, Narrow-band imaging; WOS, White opaque substance; DL, Demarcation line; EUS, Endoscopic ultrasound. The corrected p-values were adjusted using the Benjamini-Hochberg method.

### Differences in characteristics between the complete and incomplete endoscopic papillectomy of ampullary adenomas

Overall, complete resection of ampullary adenomas was achieved in twenty-four patients (29/40, 72.5%). Five patients were referred for surgery and six patients were referred for further endoscopic treatments (such as argon plasma coagulation or radiofrequency ablation) after incomplete endoscopic resection. Normal levels of ALT and GCT were significantly associated with complete endoscopic papillectomy. Tumor extension into the bile duct under EUS, width of lower bile duct under EUS, were also associated with incomplete endoscopic papillectomy. Tumor size, submucosal injection, type of resection, and number of preoperative biopsies did not show a significant association with complete endoscopic papillectomy. Following the implementation of the Benjamini-Hochberg method for multiple comparisons, the significant findings retained their significance (Table 3).

**Table 3:** Comparative differences between complete and incomplete endoscopic papillectomy of ampullary adenomas.

Characteristics	Complete (N = 29)	Incomplete (N = 11)	P value	Corrected p-values
Age	57.6	62.7	0.27	/
Gender			0.19	/
Male	19(65.5%)	4(36.4%)		
Female	10(34.5%)	7(63.6%)		
ALT			0.04	**0.04**
Normal	26(89.7%)	6(54.5%)		
Elevate	3(10.3%)	5(45.5%)		
AST			0.10	/
Normal	28(96.6%)	8(72.7%)		
Elevate	1(3.4%)	3(27.3%)		
ALP			0.07	/
Normal	27(93.1%)	7(63.6%)		
Elevate	2(6.9%)	4(36.4%)		
GGT			0.01	**0.02**
Normal	24(82.8%)	4(36.4%)		
Elevate	5(17.2%)	7(63.6%)		
Total bilirubin			0.6	/
Normal	25(86.2%)	8(72.7%)		
Elevate	4(13.8%)	3(27.3%)		
CA199			0.99	/
Normal	26(89.7%)	10(90.9%)		
Elevate	3(10.3%)	1(9.1%)		
Erosion and redness of papilla			0.7	/
Yes	7(24.1%)	4(36.4%)		
No	22(75.9%)	7(63.6%)		
Morphological classification			0.7	/
Intra-ampullary	3(10.3%)	2(18.2%)		
Periampullary	14(48.3%)	4(36.4%)		
Hybrid	12(41.4%)	5(45.5%)		
Tumor extends into the bile duct under EUS			0.02	**0.03**
Yes	1(3.4%)	4(36.4%)		
No	28(96.6%)	7(63.6%)		
Width of lower bile duct under EUS (cm)	0.60	1.12	0.01	**0.02**
Tumor size under EUS (cm)	1.6	1.9	0.14	/
Submucosal injection			0.99	/
Yes	14(48.3%)	5(45.5%)		
No	15(51.7%)	6 (54.5%)		
Type of resection			0.99	/
En bloc resection	24(82.8%)	9(81.8%)		
Piecemeal resection	5(17.2%)	2(18.2%)		
Number of preoperative biopsies	4.0	3.82	0.67	/

ALT, Alanine transaminase; AST, Aspartate transaminase; ALP, Alkaline phosphatase; GGT, Gamma-glutamyl transferase; CA199, Carbohydrate antigen 19−9; EUS, Endoscopic ultrasound. The corrected p-values were adjusted using the Benjamini-Hochberg method.

### Differences in characteristics among patients with and without complications after endoscopic papillectomy

Complications occurred in 11 patients during our study (11/40, 27.5%). Acute mild pancreatitis occurred in six patients (6/11, 54.5%) and was successfully managed with conservative treatment. Post-procedural hemorrhage developed in four patients and was controlled by endoscopic management (4/11, 36.4%). One patient suffered from hemorrhage and perforation and was subsequently treated with endoscopic management and antibiotics. No significant differences in complications were observed based on age, gender, presence of a diverticulum, tumor extension into the bile duct under EUS, tumor sizes under EUS, submucosal injections, types of resection, placement of duct stents, or procedure duration (Table 4).

**Table 4:** Comparative differences among patients with and without complications after endoscopic papillectomy.

Characteristics	Complications (N = 11)	No complications (N = 29)	P value
Age	59.3	58.1	0.8
Gender			0.3
Male	5	18	
Female	6	11	
Presence of diverticulum			0.15
Yes	5	5	
No	6	24	
Tumor extends into the bile duct under EUS			0.99
Yes	1	4	
No	10	25	
Tumor size under EUS (cm)	1.7	1.7	0.99
Submucosal injection			0.2
Yes	3	16	
No	8	13	
Type of resection			0.99
En bloc resection	8	19	
Piecemeal resection	2	5	
Placement of pancreatic stents			0.3
Yes	10	20	
No	1	9	
Procedure time (min)	30.9	20.9	0.11

EUS, Endoscopic ultrasound.

## Discussion

In this retrospective, single-center cohort study, various characteristics were compared between individuals with and without pathological upgrades of ampullary adenomas. Treatment of the patients’ ampullary adenomas involved both surgery and endoscopic resection. The current European Society of Gastrointestinal Endoscopy (ESGE) guidelines suggest that endoscopic papillectomy should be conducted in patients identified with an ampullary adenoma with intraductal extension not surpassing 2 cm [23]. In cases where adenocarcinoma is not yet confirmed, ESGE does not recommend diagnostic or therapeutic papillectomy [23]. Evaluating ampulla lesions and accurately distinguishing between benign and malignant ones is crucial. Obtaining a histological diagnosis of the suspicious lesion through preoperative biopsy is essential for guiding treatment decisions. Endoscopic features like ulceration, friability, and spontaneous bleeding are typically linked to malignant lesions [25]. Nonetheless, relying solely on endoscopic assessment of tumor appearance is insufficient for distinguishing between benign and malignant lesions; a conclusive histological diagnosis is imperative. Nevertheless, the reliability of preoperative biopsies remains a topic of debate in clinical practice. A high rate of false negatives was noted in previous studies on preoperative lesion diagnoses, suggesting a tendency for underestimation [22,26,27]. It seemed that underestimating the preoperative diagnosis was prevalent, potentially attributed to insufficient sampling and failure to identify adenocarcinoma foci within adenomatous tissue.

In the present study, we observed a pathological upgrading rate of 42.5% (17/40) among patients, underscoring a significant prevalence of false negatives in forceps biopsy specimens. Our results align with previous reports and offer further backing for the inclusion of diagnostic EP in specific cases, irrespective of the absence of evident malignancy in the initial biopsy. Identifying distinctive clinical or endoscopic features to categorize high-risk ampullary adenomas could improve the decision-making process for choosing suitable treatment strategies. Elevated ALT and ALP levels, erosion, papillary redness, a hybrid histological type, extended lower bile duct width, and prolonged procedure duration were significantly correlated with pathological upgrading in the current study. Some significant findings retained their significance following the application of the Benjamini-Hochberg method for multiple comparisons. It is essential to underscore that our results are preliminary and exploratory, underscoring the need for validation in larger, multi-center prospective cohorts. We achieved a complete adenoma resection rate of approximately 73.5%, consistent with a prior study [18]. Endoscopic papillectomy was found to be safe in a recent retrospective study and demonstrated lower R0 rates compared to transduodenal surgical ampullectomy [20]. Historically, the extension of ampullary adenomas into the biliary duct has posed a challenge for endoscopic management [15,16,28]. Our study found a correlation between tumor extension into the bile duct with incomplete endoscopic papillectomy. This association remained significant even after applying the Benjamini-Hochberg method for multiple comparisons. Several studies have validated a reduction in procedure-related complications during the endoscopic treatment of ampullary adenomas. Onkendi et al. reported findings from a large comparative study on the outcomes of operative versus endoscopic resection, revealing a 29% incidence of post-endoscopic complications [11]. In our cohort, procedure-related complications occurred in 27.5% of patients. These complications predominantly included bleeding, perforations, and pancreatitis. All endoscopic papillectomy-related complications were effectively managed using conventional methods, and no severe complications were noted in this study. The presence of a diverticulum did not show a significant correlation with the occurrence of complications, aligning with previous research; however, further investigation would be required for validation [29].

There were several potential limitations to our study. First, the small sample size increased the risk of both false positives and false negatives in our exploratory study. To mitigate the issue of multiple testing, we applied Bonferroni corrections for specific comparisons. Second, the study aimed to explore the relationship between clinical and endoscopic variances and pathological upgrade and incomplete resection. However, due to the lack of univariable and multivariable modeling, no risk factors could be identified for predicting pathological upgrade and incomplete resection. Third, pathological upgrading cases, encompassing transitions from LGD to HGD and from HGD to adenocarcinoma, were consolidated into a unified outcome group. Despite recognizing distinct biological behaviors and clinical consequences among these sub-types, the constraints of event numbers precluded subgroup analysis. Fourth, the possibility of selection and recall bias was inevitable due to the retrospective nature of this study. Strict inclusion and exclusion criteria were applied in this study to adhere to practice guidelines and ensure procedural safety. As a result, the findings are most relevant to early-stage, non-FAP-related ampullary adenomas suitable for endoscopic resection. This limits the generalizability of the results to patients with advanced disease, significant ductal involvement, or syndromic polyposis conditions like FAP, who typically opt for surgical management. Further large-scale prospective studies are needed to overcome these limitations.

In conclusion, our study suggests that pre-procedural biopsy of ampullary lesions may not provide a comprehensive representation of their characteristics. Therefore, it is crucial to closely monitor the possibility of pathological upgradation in patients with elevated levels of ALT and ALP, erosion and redness of the papilla, hybrid histological type, and wider lower bile ducts identified during EUS examination. Additionally, we have substantiated the safety and effectiveness of endoscopic papillectomy for ampullary lesions when conducted by experienced endoscopists.

## Supporting information

S1. Data set (CSV)
